# A case of progressive giant coronary aneurysm following first-generation drug-eluting stent implantation for chronic total occlusion-a case report and literature review

**DOI:** 10.3389/fcvm.2026.1787900

**Published:** 2026-03-20

**Authors:** Bin Li, Shufang Han, Zhen Li, Qun Jin

**Affiliations:** Department of Cardiology, The 960th Hospital of The Joint Logistics Support Force, Jinan, China

**Keywords:** acute myocardial infarction, coronary artery aneurysm, coronary artery bypass grafting, drug-eluting stent, stent complication

## Abstract

Coronary artery aneurysm (CAA) is a segmental dilation of the coronary artery and a rare condition. The incidence of CAA after drug-eluting stent (DES) implantation is 0.2%–2.3%, with giant coronary artery aneurysm (GCAA) being even rarer, occurring in approximately 0.02% of cases. We report a case of a 46-year-old female who presented with paroxysmal precordial pain in 2010. Coronary angiography was performed, and four sirolimus-eluting stents (FireBird) were implanted. Over the following decade, she experienced multiple acute coronary ischemic events. Repeat angiography revealed that coronary aneurysms had developed at all original stent implantation sites and were progressively enlarging. The patient ultimately underwent surgical treatment and recovered well during one year of follow-up. This rare case of stent-related GCAA suggests that treatment decisions should comprehensively evaluate the patient's specific conditions, including the recurrence of thrombotic events, the efficacy and safety of antithrombotic therapy, as well as the location and size of the aneurysm.

## 1 Background

Drug-eluting stents (DES) used for percutaneous coronary intervention (PCI) consist of a metallic stent scaffold, a polymer acting as a drug carrier, and an anti-restenotic drug embedded within the polymer. All commercially available DES are based on the same fundamental components but differ in alloy platform, polymer type, and the anti-restenotic drug used (1).

Firebird DES, approved for marketing in China in 2004 by MicroPort Scientific Co., Ltd., is a first-generation DES. Its platform is made of 316L stainless steel with a strut thickness of 140–150 micrometers. The durable polymer is composed of two layers: an outer layer of poly(butyl methacrylate) and an inner layer of poly(ethylene-vinyl acetate). Sirolimus is mixed into the polymer, with over 80% of the drug released within 30 days and the remaining drug released slowly over a total release cycle of approximately 90 days.

A coronary artery aneurysm (CAA) is defined when the coronary artery diameter exceeds 50% of the diameter of the adjacent normal coronary artery segment (2). A CAA can be termed a giant coronary artery aneurysm (GCAA) if its diameter exceeds four times that of the normal segment or is directly greater than 20 mm, although there is no definitive consensus on this definition (3).

The incidence of CAA after DES implantation is 0.2%–2.3%, representing a rare complication, with GCAA being even rarer at an incidence of 0.02%. The underlying mechanisms remain unclear, but several hypotheses have been proposed. Deep arterial wall injury during stent implantation and high-pressure post-dilation balloon inflation are considered causes of aneurysm formation. Furthermore, as noted by Aoki et al. (4) in their review, the antiproliferative effect of sirolimus is also clearly associated with aneurysm formation. The localized release of antiproliferative drugs from DES, while effectively inhibiting neointimal hyperplasia, may also delay vascular healing after injury, theoretically increasing the risk of CAA formation. Additionally, alterations in the vascular wall environment at the DES implantation site are believed to be important contributors to CAA development, including delayed endothelialization, medial inflammatory reactions, and hypersensitivity to the drug or polymer mixture (4–6). In particular, polymers have been shown to provoke significant inflammatory reactions, leading to eosinophilic/heterophilic cell infiltration into the vessel wall. This view is supported by multiple animal studies and human autopsy studies (4, 7, 8). These changes in the arterial wall environment are thought to lead to vessel wall destruction and weakening, thereby triggering dilation and promoting aneurysm formation (5, 6). Although coronary aneurysms are often asymptomatic and may even regress spontaneously, they are associated with adverse clinical events; both thrombosis and aneurysm rupture are life-threatening problems.

## 2 Case presentation

This case report details the clinical course of a 46-year-old female patient with a history of hypertension, managed with long-term oral captopril and achieving well-controlled blood pressure. She had no history of smoking or alcohol abuse, nor a family history of premature coronary artery disease. The patient was first admitted in April 2010 due to “unstable angina.” On admission, her vital signs were stable, and physical examination revealed no positive findings. The pre-procedural electrocardiogram showed significant horizontal ST-segment depression in leads V1-V6. Echocardiography revealed a left ventricular ejection fraction (LVEF) of 55% with no regional wall motion abnormalities at rest. Cardiac enzymes and troponin were negative.

**Figure 1:**
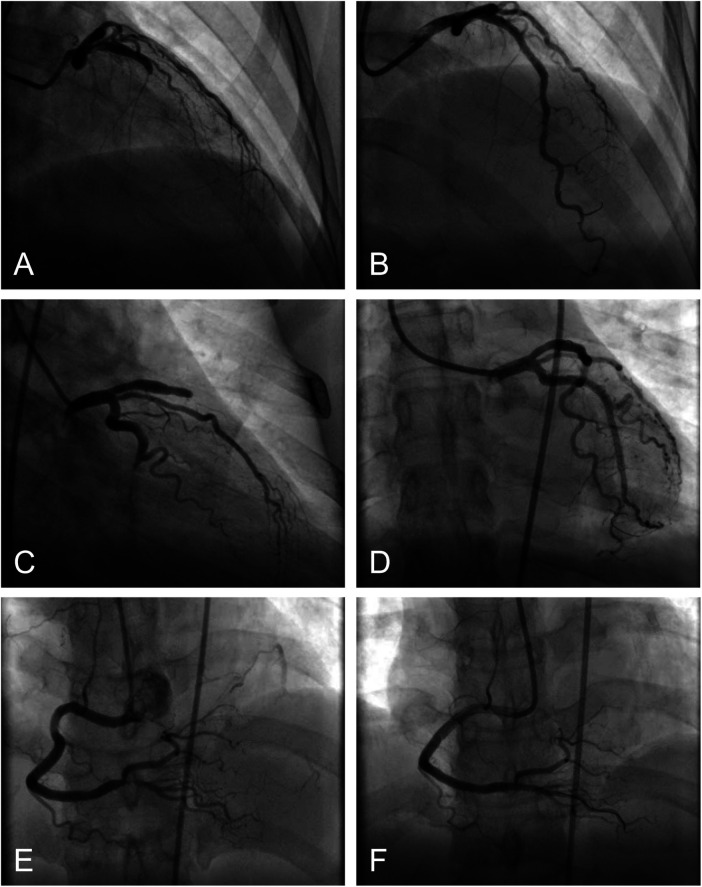
PCI procedure in 2010; **(A,C,E)**: Pre-procedural status of LAD, LCX, and RCA; **(B,D,F)**: Status of LAD, LCX, and RCA after stent implantation.

Coronary angiography (CAG) was subsequently performed with the following findings: The left anterior descending artery (LAD) was completely occluded proximally (Figure 1A); the left circumflex artery (LCX) was completely occluded distally (Figure 1C); the right coronary artery (RCA) was well-developed with 70% segmental stenosis in its proximal segment and 90% segmental stenosis at the ostium of the posterior descending artery (PDA) (Figure 1E). The RCA provided collateral circulation to the distal LAD and LCX.

Based on comprehensive imaging and clinical data, the LAD and LCX were considered chronic total occlusions (CTOs) with collaterals supplied by the RCA. However, collateral flow volume was significantly less than antegrade flow, functionally equivalent to severe stenosis, consistent with the extensive anterior wall ischemia indicated by the ECG. Additionally, the RCA itself had severe stenosis, further exacerbating myocardial ischemia. Therefore, the RCA was identified as the culprit vessel for the presenting symptoms. Given the patient's relatively young age and preserved cardiac function (LVEF 55%), it was decided to pursue complete revascularization. For this patient with multi-vessel disease (SYNTAX score 23–32), the score falls within the intermediate-risk category, wherein both revascularization strategies (PCI and CABG) are considered acceptable according to contemporary guidelines. Within this therapeutic equipoise, the final treatment selection was guided by several patient-specific factors. First, the patient's young age raised critical concerns regarding long-term saphenous vein graft patency. Preserving native vessels for potential future CABG by avoiding premature graft implantation was a strategic consideration, as vein grafts in young patients have accelerated failure rates over their decades-long life expectancy. Second, the patient and her family expressed strong concerns regarding the trauma of open-heart surgery, including sternotomy, prolonged recovery, and potential perioperative risks. This preference for a less invasive approach was respected. Ultimately, we adopted the multi-vessel interventional strategy described above.

Subsequently, a 3.0 mm × 29 mm FireBird stent was implanted in the proximal LAD(Figure 1B); a 2.5 mm × 18 mm FireBird stent was implanted in the distal LCX (Figure 1D); a 3.5 mm × 18 mm FireBird stent was implanted in the proximal RCA; and a 2.5 mm × 18 mm FireBird stent was implanted at the PDA ostium(both shown in Figure 1F). The procedure was successful. The patient was discharged on dual antiplatelet therapy (aspirin and clopidogrel) and simvastatin for lipid management. During follow-up at our hospital (3, 6, and 12 months post-procedure), the patient remained asymptomatic, with no significant dynamic changes on ECG or echocardiography. Lipid levels were controlled below 1.8 mmol/L, and liver and kidney function were normal. After one year of follow-up at our hospital, she continued follow-up at a local hospital.

**Figure 2:**
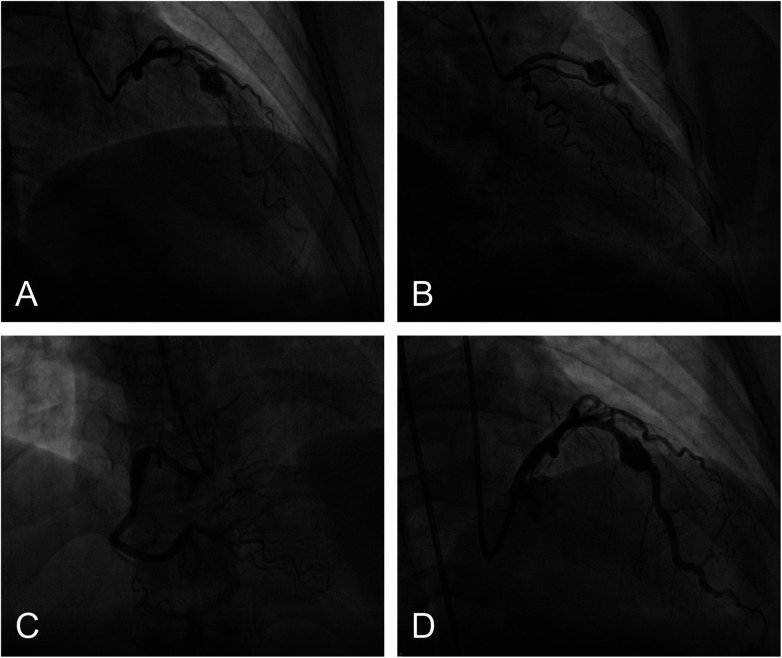
Coronary angiography showing aneurysm formation in the LAD and RCA; **(A,B)**: LCX occlusion, LAD in-stent restenosis with aneurysm formation; **(C)**: Aneurysm formation at the RCA stent site; **(D)**: Status after re-stenting of the LAD.

Seven years later (May 2017), the patient presented to a local hospital with “chest pain and diaphoresis for 3 h.” Admission ECG showed sinus rhythm with ST-segment depression in leads V1-V6, and troponin T was 0.173 ng/mL (reference value 0-0.014). A diagnosis of NSTEMI was made, and coronary angiography was performed. CAG showed 95% focal in-stent restenosis accompanied by obvious coronary aneurysm formation in the proximal LAD stent, with slowed distal LAD flow (TIMI grade 2). The LCX was completely occluded at the ostium of the original stent. Aneurysmal dilation was noted at the proximal RCA stent site, and there was 95% segmental stenosis at the ostium of the posterior left ventricular branch (PLA) (Figures 2A–C). An attempt to recanalize the LCX failed. Considering the LCX was a chronic total occlusion (CTO) and the culprit vessel was the LAD, a 3.0 mm  ×  15 mm Resolute stent was implanted within the existing LAD stent with a good visual result (Figure 2D). Post-procedure ECG showed sinus rhythm with T-wave inversion in leads V1-V6. Echocardiography revealed no significant structural abnormalities and an LVEF of 63%. The patient was discharged on continued dual antiplatelet therapy (aspirin and clopidogrel) and atorvastatin, with regular follow-up.

**Figure 3:**
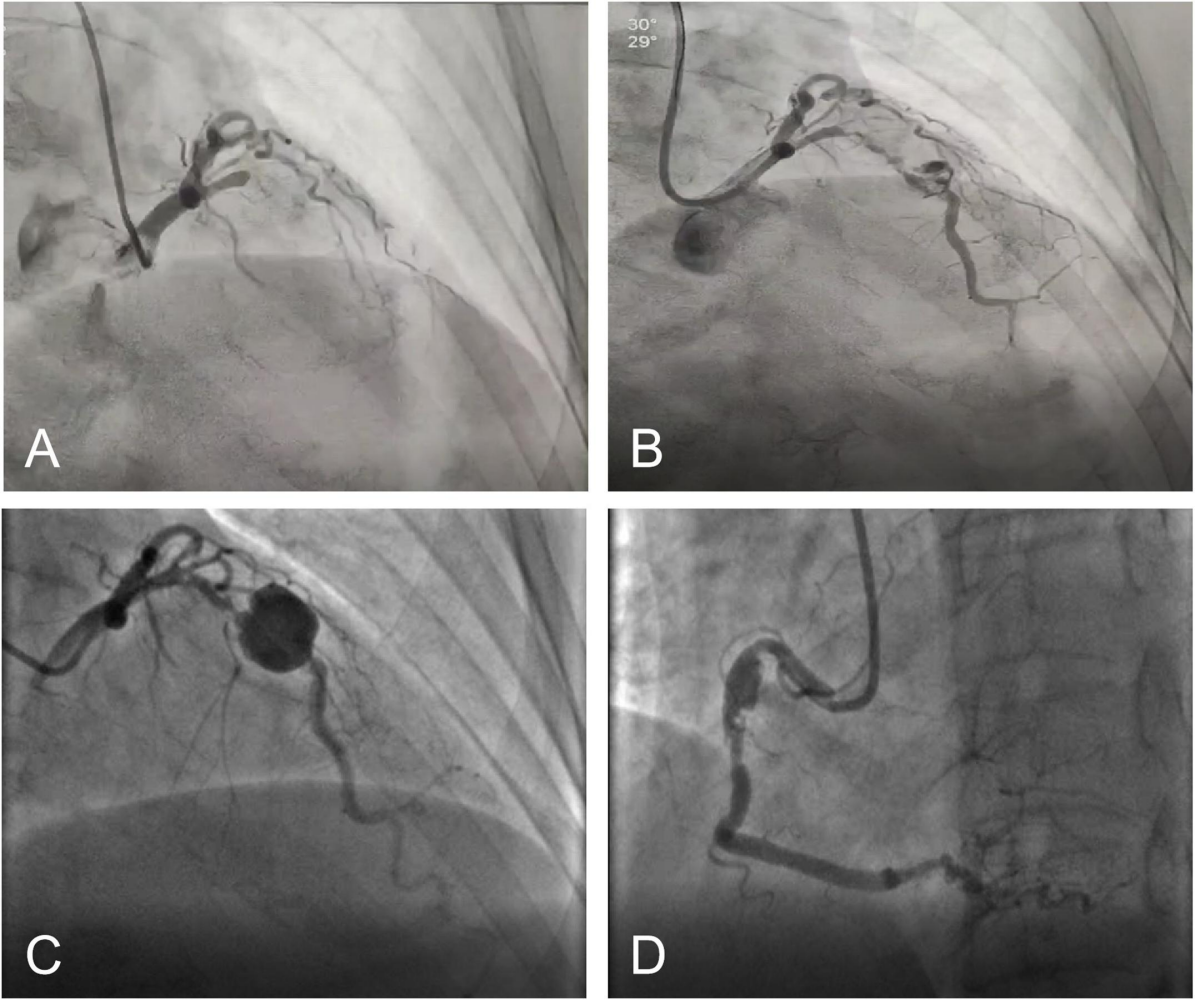
Two coronary interventions in 2024. **(A)**: Emergency CAG showing occlusion of the proximal LAD; **(B)**: Restoration of flow after intracoronary thrombolysis and thrombus aspiration; **(C,D)**: Follow-up CAG after 3 weeks of antithrombotic therapy.

Fourteen years later (December 2024), the patient again presented to a local hospital with “chest pain and diaphoresis for 4 h.” Admission ECG showed sinus rhythm with significant ST-segment depression. Troponin T was 0.045 ng/mL (reference value 0-0.014). A diagnosis of NSTEMI was made, and emergency PCI was performed. Initial CAG showed: complete occlusion of the proximal LAD, occlusion within the distal LCX stent (distal flow TIMI grade 2); the aneurysm in the proximal RCA had progressed significantly compared to previous imaging, with 70% in-stent stenosis in the proximal segment and subtotal occlusion of the PLA (Figure 3A). After guidewire passage and balloon pre-dilation of the LAD lesion, a giant aneurysm with heavy thrombus burden was found in the proximal LAD. Thrombus aspiration and intracoronary injection of 100,000 units of urokinase restored TIMI grade 3 flow (Figure 3B). Post-procedure ECG showed sinus rhythm with deep T-wave inversion. Echocardiography revealed left ventricular filling abnormalities but no significant structural abnormalities, with an LVEF of 55%. After intensified antithrombotic therapy (clopidogrel and rivaroxaban), the patient was transferred to our hospital for further management.

After completing immunological tests at our hospital to rule out autoimmune diseases, the patient continued on dual antiplatelet therapy (aspirin and ticagrelor) for three weeks. Repeat CAG showed: 70%–90% stenosis within the proximal LAD stent, with significant aneurysmal dilation distal to the stent measuring 15 mm × 17 mm, indicating clear progression. The distal LCX had subtotal occlusion with distal flow TIMI grade 1–2. Significant aneurysmal dilation was seen at the mid RCA stent site, measuring approximately 5 mm × 10 mm, with 70%–80% in-stent stenosis. Mild aneurysmal dilation was also noted at the distal RCA stent site, and there was 95% segmental stenosis at the PLA ostium (Figures 3C,D). The heart team evaluated the patient and concluded that the size and morphology of the aneurysms were unsuitable for PCI. Given the clear association between the aneurysms and prior PCI procedures, along with recurrent thrombosis at the LAD aneurysm site, surgical treatment was recommended. After discussing the risks of conservative management and the limitations of percutaneous intervention with the patient and her family, surgical treatment was decided upon. During thoracotomy, the giant proximal LAD aneurysm was found to be adherent and organized to surrounding tissues. Therefore, ligation of the aneurysm followed by bypass grafting was performed. The patient's postoperative recovery was satisfactory, during one year of follow-up, there were no dynamic changes on ECG, no segmental wall motion abnormalities on echocardiography, and the ejection fraction remained above 50%. She experienced no episodes of chest tightness or pain. A chronological summary of all procedures, implanted stents, and key clinical events is presented in Table 1.

**Table 1:** Chronological summary of all interventional procedures, implanted stents, key clinical events, and antithrombotic therapy throughout the 14-year follow-up period.

Date	Indication	Intervention Site	Stent Type/Size or Procedure	Key Events/Outcomes	Antithrombotic Therapy
Apr 2010	Unstable Angina	Proximal LAD	FireBird (3.0 × 29 mm)	Initial PCI; no evidence of aneurysm	Aspirin + Clopidogrel
		Distal LCX	FireBird (2.5 × 18 mm)		
		Proximal RCA	FireBird (3.5 × 18 mm)		
		PDA Ostium	FireBird (2.5 × 18 mm)		
May 2017	NSTEMI	Proximal LAD	Resolute (3.0 × 15 mm)	First detection of aneurysms (LAD and RCA sites)	Aspirin + Clopidogrel
Dec 2024	NSTEMI	Proximal LAD	Thrombus aspiration + urokinase	Giant aneurysm (15 × 17 mm) with heavy thrombus burden	Clopidogrel+Rivaroxaban
Dec 2024	Elective PCI	Proximal LAD	No intervention; CABG decided	Aneurysm size/morphology unsuitable for PCI	Low Molecular Weight Heparin
Dec 2024	Multi-vessel disease + GCAA	LAD,RCA	Aneurysm ligation + CABG	Successful surgical treatment	Aspirin + Ticagrelor

## 3 Discussion

This patient developed stent-related coronary aneurysms at multiple sites. The largest aneurysm, located in the proximal LAD, exceeded four times the diameter of the adjacent segment, meeting the diagnostic criteria for GCAA. Based on shape, CAAs are classified as saccular if the longitudinal diameter is less than the transverse diameter, and fusiform if the opposite is true. However, data on the prognostic significance of aneurysm shape are insufficient (3). Both aneurysms in this case were fusiform. As noted by Pala et al. (9), although coronary angiography is a common tool for diagnosing CAA, the precise assessment of the lumen can often be compromised by slow flow or contrast agent stasis within the aneurysmal segment. In such circumstances, intravascular ultrasound (IVUS) can provide crucial Supplementary Information, such as clarifying the true size of the aneurysm, identifying mural thrombus, and assessing vessel wall remodeling. However, for this patient, it was considered that IVUS would not alter the treatment strategy, so it was not performed for economic reasons.

The aneurysms in this patient were closely associated with stent implantation sites. Several mechanisms may explain this relationship. First, the antiproliferative effect of sirolimus may play a role. While effectively inhibiting neointimal hyperplasia, localized drug release can also delay vascular healing after injury, potentially increasing CAA risk.Second, hypersensitivity and inflammatory reactions to the stent polymer are likely contributors. Although stent polymers are highly biocompatible, rare cases of severe local inflammation have been reported. Such reactions, characterized by eosinophil and lymphocyte infiltration involving all three arterial layers, can lead to aneurysm formation. It has also been reported that CTO lesions may have some correlation with aneurysm formation (10). Due to the complexity of CTO lesions, there is a higher likelihood of damaging the elastic components of the media during PCI, leading to gradual luminal enlargement (especially with high-pressure post-dilation after stent implantation), vessel wall thinning, and remodeling, ultimately resulting in CAA formation. In this patient, the LAD and LCX were chronic total occlusions. However, an aneurysm also developed at the RCA stent site, which was not an occlusive lesion. Furthermore, the LAD aneurysm was significantly larger than the RCA aneurysm. Therefore, while the underlying lesion characteristics likely played a role, they were not the dominant factor in aneurysm formation in this patient.

The patient received a total of four FireBird stents from MicroPort and one Resolute stent from Medtronic. The four FireBird stents were implanted in 2010, with no signs of aneurysm formation prior to implantation. In 2017, follow-up CAG for NSTEMI revealed stent-related coronary aneurysms in the LAD and RCA, and another Resolute stent was implanted within the existing FireBird stent in the LAD. The 2024 CAG showed progression in the size of these aneurysms, with the LAD aneurysm showing particularly significant enlargement to 15 × 17 mm. Since the second Resolute stent was implanted inside the original stent, it is impossible to determine whether aneurysm formation in this case was related to the specific type of stent.

Due to the rarity of GCAA and the lack of randomized controlled trials, treatment strategies remain controversial. Hong et al (11). followed patients with CAA and myocardial infarction treated with DES and found improved clinical outcomes. However, they also noted limitations: the inability of graft materials to endothelialize may lead to chronic platelet activation, increasing risks of thrombosis, in-stent restenosis, distal embolization, stent malposition, coronary dissection, and rupture.Therefore, for large GCAA with a high risk of rupture and myocardial infarction, coronary artery bypass grafting (CABG) is considered necessary and preferable (2, 3, 12). Consistent with the view of Pala et al. (9), surgical intervention should be prioritized for multiple giant coronary aneurysms to prevent fatal complications such as long-term rupture or embolization.Multicenter studies suggest that CABG is the optimal choice for patients with atherosclerosis-related giant CAA and acute myocardial infarction (13, 14). Surgical intervention may include aneurysm resection, ligation, with or without bypass grafting.

In this case, the heart team explored all treatment options. The patient had been on long-term regular antiplatelet therapy but still experienced recurrent GCAA-related myocardial infarction with suboptimal results. The addition of oral anticoagulation did not guarantee efficacy while carrying a higher risk of bleeding. Since the aneurysms were clearly associated with prior stent implantation, further stent implantation was contradictory to the treatment goal. This patient presented with multiple-vessel GCAA on angiography and recurrent CAA-related myocardial infarction, meeting the criteria suggested by multicenter studies for CABG. Therefore, the heart team decided on surgical treatment, which yielded favorable therapeutic results.

## 4 Conclusion

In conclusion, CAA formation after DES implantation is a rare complication that may be underestimated due to its often asymptomatic nature. The exact mechanism of CAA formation post-DES implantation remains unclear, but stent polymers likely participate in the inflammatory response. There is no consensus on the treatment of GCAA. Treatment decisions should be tailored to the specific situation, considering factors such as the recurrence of thrombotic events, the efficacy and safety of antithrombotic therapy, and the location and size of the aneurysm. Long-term follow-up is recommended for patients who develop CAA after DES implantation.

## Data Availability

The original contributions presented in the study are included in the article/Supplementary Material, further inquiries can be directed to the corresponding author.
